# Hand, Foot, and Mouth Disease Caused by Coxsackievirus A6, Japan, 2011

**DOI:** 10.3201/eid1802.111147

**Published:** 2012-02

**Authors:** Tsuguto Fujimoto, Setsuko Iizuka, Miki Enomoto, Katsuhiko Abe, Kazuyo Yamashita, Nozomu Hanaoka, Nobuhiko Okabe, Hiromu Yoshida, Yoshinori Yasui, Masaaki Kobayashi, Yoshiki Fujii, Hiroko Tanaka, Miwako Yamamoto, Hiroyuki Shimizu

**Affiliations:** National Institute of Infectious Diseases, Tokyo, Japan (T. Fujimoto, K. Yamashita, N. Hanaoka, N. Okabe, H. Yoshida, Y. Yasui, H. Shimizu);; Shimane Prefectural Institute of Public Health and Environmental Science, Shimane, Japan (S. Iizuka);; Hyogo Prefectural Institute of Public Health and Consumer Sciences, Hyogo, Japan (M. Enomoto);; Hiroshima City Institute of Public Health, Hiroshima, Japan (K. Abe, Y. Fujii, H. Tanaka, M. Yamamoto);; Kobayashi Pediatric Clinic, Shizuoka, Japan (M. Kobayashi)

**Keywords:** enterovirus, hand, foot, and mouth disease, herpangina, coxsackievirus, coxsackievirus A6, onychomadesis, expedited, Japan

**To the Editor:** Coxsackievirus A6 (CVA6) belongs to human enterovirus species A of the genus *Enterovirus*. According to a Japanese Infectious Agents Surveillance Report, this virus is one of the major causes of herpangina, an acute febrile disease characterized by vesicles, ulcers, and redness around the uvula, which occurs mainly in young children and infants (*1*).

In June 2011, a sudden increase in cases of hand, foot, and mouth disease (HFMD) at pediatric sentinel sites (≈3,000 pediatric hospitals and clinics) was reported to the National Epidemiologic Surveillance of Infectious Diseases System in Japan. Compared with past numbers of cases over 30 years of surveillance, the number of cases of HFMD per sentinel site peaked in week 28 (July) of 2011 (10.97 cases per sentinel), particularly in western Japan (*2*). According to the Infectious Agents Surveillance Report (as of September, 18, 2011), CVA6 was detected in 709 HFMD cases and 156 herpangina cases throughout Japan (*1*).

Clinical samples (throat swab specimens and feces) obtained from sentinel sites in Shimane, Hyogo, Hiroshima, and Shizuoka, Japan, were screened for enteroviruses by using an enterovirus-specific reverse transcription PCR and sequence analysis of the partial viral protein (VP)4/VP2 or VP1 region (*3*). Among 93 clinical samples from 108 HFMD case-patients, we identified 74 case-patients as CVA6 positive by sequence analysis.

On the basis of sequence analysis of the entire VP1 region (GenBank accession nos. AB649286–AB649291), the consensus sequence had 82.3%–82.5% nt identity (94.8%–95.4% aa identity) with the prototype CVA6 Gdula strain (GenBank accession no. AY421764). CVA6 was not isolated from clinical samples in a cell culture system. Therefore, most CVA6 strains were identified by molecular detection directly from clinical samples and sequence analysis. Some CVA6 strains were grown and isolated in suckling mice; these strains were antigenically identified as CVA6 by a neutralization test with specific antiserum against CVA6 (*4*).

In Japan, HFMD and herpangina are classified as category V infectious diseases. On the basis of clinical diagnosis, suspected infections were reported by pediatric sentinel sites on a weekly basis to the Infectious Disease Surveillance Center of the National Institute of Infectious Diseases (Tokyo, Japan). Typical clinical signs and symptoms of HFMD cases caused by CVA6 were fever, mild vesicles in oral mucosa, and skin blisters on hands, arms, feet, legs, buttocks, and nail matrixes (Figure). Some patients with HFMD had onychomadesis (periodic shedding of the nails) 1–2 months after onset of HFMD. Most cases of HFMD were self-limited. However, additional follow-up may be necessary for patients with onychomadesis who are treated at dermatology clinics.

**Figure:**
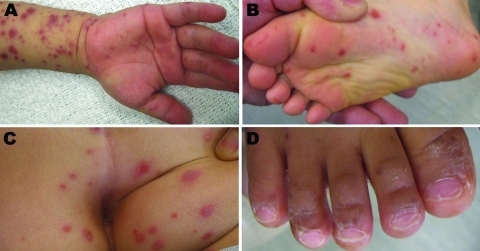
Typical clinical manifestations of hand, foot, and mouth disease associated with coxsackievirus CVA6 in Shizuoka, Japan, June–July, 2011. A) Hand and arm of a 2.5-year-old boy; B) foot and C) buttocks of a 6-year-old boy; D) nail matrix of a 20-month old boy.

As in other countries in the Asia–Pacific region, major causes of HFMD in Japan were CVA16 and enterovirus 71. In 2010, enterovirus 71 was identified as a major cause of HFMD (*2*). In contrast, CVA6 was consistently associated with herpangina, as were CVA2, CVA4, CVA5, and CVA10, but CVA6 was occasionally detected in HFMD case-patients. CVA6 was the major cause of herpangina in 2007, but an increase in the detection rate of CVA6 in HFMD case-patients was reported in Japan in 2009 (*2*).

HFMD outbreaks caused by CVA6 were reported in Singapore, Finland, and Taiwan in 2007–2009 (*5**–**8*). Recent HFMD outbreaks in Finland and Spain were associated with cases of onychomadesis 1–2 months after onset of HFMD (*6**,**8**,**9*). In Japan, cases of onychomadesis after onset of HFMD were reported in 2009 (*10*). Therefore, changes in clinical outcomes of CVA6-associated diseases should be investigated.

Although most HFMD cases caused by CVA6 in Japan were mild, CVA6 was also detected in other clinical samples, including cerebrospinal fluid from a patient with acute encephalitis in Hiroshima, which reaffirmed possible additional clinical manifestations during an HFMD outbreak caused by CVA6. Careful surveillance of disease and infectious agent activities are crucial in monitoring CVA6-associated HFMD, onychomadesis, and neurologic diseases. Nucleotide identity between CVA6 strains in Finland (2008) (*7*) and Japan (2011) was ≈95% in the partial VP1 region. More detailed genetic, phenotypic, and epidemiologic analyses of CVA6 are needed to determine the role of CVA6 in HFMD outbreaks with or without onychomadesis.
